# A scoping review of AI in the classification of oral cancer and oral potentially malignant disorders from visible-light photography

**DOI:** 10.1177/20552076261481593

**Published:** 2026-08-22

**Authors:** Charles Goodmaker, Rishi Bhandari, Anwar Tappuni, Tuan D. Pham

**Affiliations:** 1 Oral and Maxillofacial Surgery, Barts Health NHS Trust, London, UK; 2 Barts and the London School of Medicine and Dentistry, 4617 Queen Mary University of London, London, UK

**Keywords:** mouth neoplasms, precancerous conditions, deep learning, image processing, computer-assisted, photography, dental, scoping review

## Abstract

**Objectives:**

To map the scope, methodological characteristics, and clinical task design of artificial intelligence (AI) models applied to visible-light photographs of the oral cavity for classification of oral cancer and oral potentially malignant disorders (OPMD).

**Methods:**

A scoping review following the Arksey and O'Malley framework and PRISMA-ScR was conducted. PubMed, Embase, Scopus, and Web of Science were searched from January 2015 to October 2025. Studies were eligible if they applied AI to intraoral photography for lesion detection, classification, or risk stratification. Data were extracted on dataset provenance, ground-truth labelling, model architecture, validation strategy, performance metrics, and reporting completeness.

**Results:**

127 studies met inclusion and were categorised by their output classes across a mutually exclusive, five-category framework (four ordered tiers plus a residual category) with a benign-OPMD-malignant disease spectrum. Over half addressed a cancer-versus-non-cancer endpoint only (54.3%, n=69), whilst 27.6% (n=35) preserved OPMD as a discrete output category. Public image datasets were used in 44.1% (n=56) of studies, and fully histologically-confirmed ground-truth labels were available in only 7.9% (n=10). External validation was reported in 8.7% (n=11), patient-level data splitting in 10.2% (n=13), and prospective clinical evaluation in 0% (n=0). ResNet-family architectures were most frequently reported (50.4%, n=64, as primary or comparator model); explainable-AI components appeared in 37.0% (n=47) and multimodal inputs in 12.6% (n=16).

**Conclusion:**

Classifying oral cancer and OPMD lesions with visible light photographs is an expanding field of research, but one which reveals space for more robust and clinically grounded training datasets as well as exploration of frontier AI methodologies.

## Introduction

Oral cancer carries significant morbidity and mortality burden. Globally there were 389,846 cases of lip and oral cavity cancer reported worldwide, making it the 16th most prevalent cancer globally.^1,2^ In England alone, in 2022/23, there were 275,354 head and neck cancer referrals to 2 week wait pathways, reflecting substantial diagnostic workload at the primary-secondary care interface, of which oral cavity lesions form an important subset.^3^

Oral cancer management, from potentially malignant lesion identification to complete treatment, requires substantial healthcare resources. For example, oral leukoplakia (OL) has been shown to progress to oral squamous cell carcinoma (OSCC), on average, in 7.2% of cases.^4^ OL can have a varied clinical appearance and be difficult to differentiate from other Oral Potentially Malignant Disorders (OPMDs) without gold standard histological biopsy. These present as significant challenges to healthcare professionals when diagnosing, monitoring and managing patients with OSCC or OPMDs. The potential impact of automated oral lesion classification and risk assessment on healthcare systems could be significant.

OPMDs are a set of oral conditions which have a higher rate of malignant transformation to OSCC, compared to benign lesions. These include oral leukoplakia, oral erythroplakia, oral submucous fibrosis (OSMF), and oral lichen planus (OLP), all of which can be associated with varying degrees of dysplasia increasing the likelihood of progression to OSCC; the most common form of oral malignancy.^5^ Therefore, like many neoplastic disorders, OSCC sits on a pathophysiological spectrum spanning benign, OPMD and malignant pathologies which can have overlapping clinical presentations.^6^ Many OPMD lesions appear similar on visual inspection alone, despite varying malignant potential, with one study citing only 25% of lesions have a provisional clinical diagnosis which matches histological outcomes.^7,8^ As a result, histopathological biopsy remains the gold standard; determining lesion type by observing architectural, cellular, genetic and biochemical transformations within tissues.^9^

**Figure 1.**
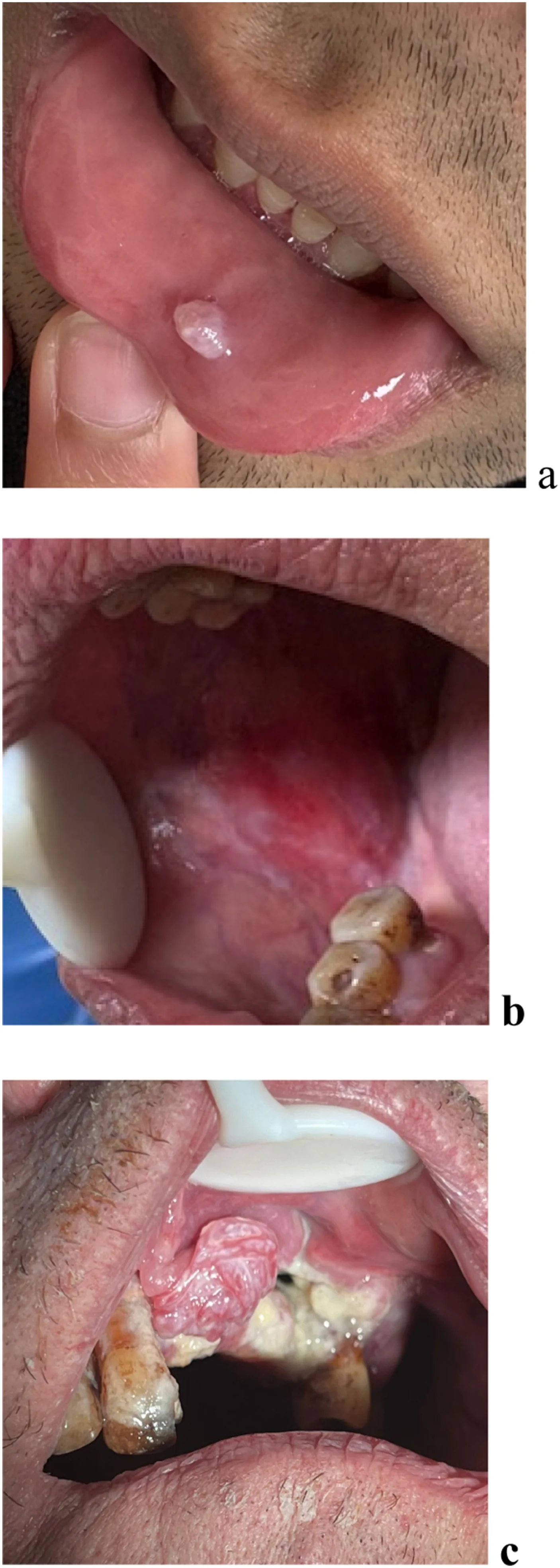
Examples of clinically distinct oral lesions: (a) a mucocele - a benign lower lip lesion; (b) a non-homogeneous red-and-white patch with high risk of dysplasia; (c) an oral squamous cell carcinoma. These photographs are representative of visible-light clinical photography acquired under real-world conditions, including the variable illumination, framing, and orientation that characterise the imaging data used in the AI literature reviewed in this study.

However, histopathology captures only a single timepoint in the disease continuum, and a single biopsy captures only one geographical area of tissue which may harbour heterogeneity which both complicates lesion monitoring and creates opportunities for early intervention before progression to advanced stages. This diagnostic challenge necessitates the integration of technological approaches that can enhance detection and risk stratification of not only benign and malignant but also precursor oral lesions (representative examples of clinically distinct oral lesions spanning benign, potentially malignant, and malignant presentations are shown in Figure 1).

Significant disparities exist in both oral cancer incidence and survival across demographic groups, with social deprivation strongly associated with higher disease rates.^1,10^ Established risk factors include tobacco smoking, alcohol consumption, betel quid chewing, and increasing age.^11,12^ Microbiological factors such as Candida infection and human papillomavirus (HPV), along with broader oral microbiome composition, have also been implicated in cancer development.^13,14^ These risk patterns, combined with unequal access to specialist diagnostic services, underscore the need for accessible diagnostic tools that can improve early detection across diverse healthcare settings.

Artificial intelligence (AI) has been shown as a promising approach in parallel specialities, such as dermatology, where the first class-IIa medical device for skin lesion classification has been deployed to address high volume referral burdens on UK clinical pathways.^15^ Similarly, AI for oral lesion classification requires consideration of clinical utility, explainability, and integration with existing workflows. AI encompasses computational systems capable of performing tasks that typically require human intelligence. Within AI, machine learning (ML) represents algorithms that learn patterns from data without explicit programming.^16^ Deep learning (DL), a subset of machine learning, employs multi-layered artificial neural networks that can automatically extract hierarchical features from raw data.^17^ In medical imaging, computer vision applies these algorithms to interpret visual information from images, enabling automated lesion detection and classification.^18^

Advances in DL architectures have led to the development of convolutional neural networks (CNNs), which have become the dominant approach for medical image classification, using specialised layers to detect spatial patterns at multiple scales.^19,20^ More recently, vision transformers have emerged as an alternative architecture that processes images as sequences of patches, enabling modelling of long-range dependencies across the entire image.^21^ These models have demonstrated competitive performance on medical imaging tasks, though their application in oral pathology remains limited.^22^

While AI methods for oral lesion analysis have been studied across a range of imaging modalities, including specialist autofluorescence, hyperspectral, and microscopy-based approaches, this review focuses on visible-light intraoral clinical photography - an imaging modality routinely collected during management of oral lesions in the UK. We map the published research activity in this domain to provide a structured view of what has been studied: the datasets used, the learning paradigms applied, the validation approaches taken, the model architectures employed, the clinical tasks addressed, and the explainability methods reported. Observations on clinical relevance and methodological practice follow on from this mapping and shed light on current feasibility of real-world and future research directions.

## Objectives

1. To map the evidence landscape of artificial intelligence in the classification and detection of oral cancer and its precursor lesions.
2. To focus on visible-light intraoral clinical photography as the primary imaging modality, while including studies that combine photographic data with textual or structured clinical information as multimodal inputs.
3. To identify gaps in research and the barriers to scaled usage in healthcare.

## Methodology

This study followed the PRISMA-ScR (scoping review extension) guidelines, a review protocol was not registered for this study.^23^ Searches were conducted on 28^th^ October 2025 in Scopus, Web of Science, Embase, and PubMed from 2015–2025 using structured Boolean strings combining terms for (1) oral lesions and cancers, (2) AI and machine learning, and (3) visible-light or intraoral imaging modalities (Supplementary File A). Radiographic and histopathology-only studies were excluded.

**Figure 2.**
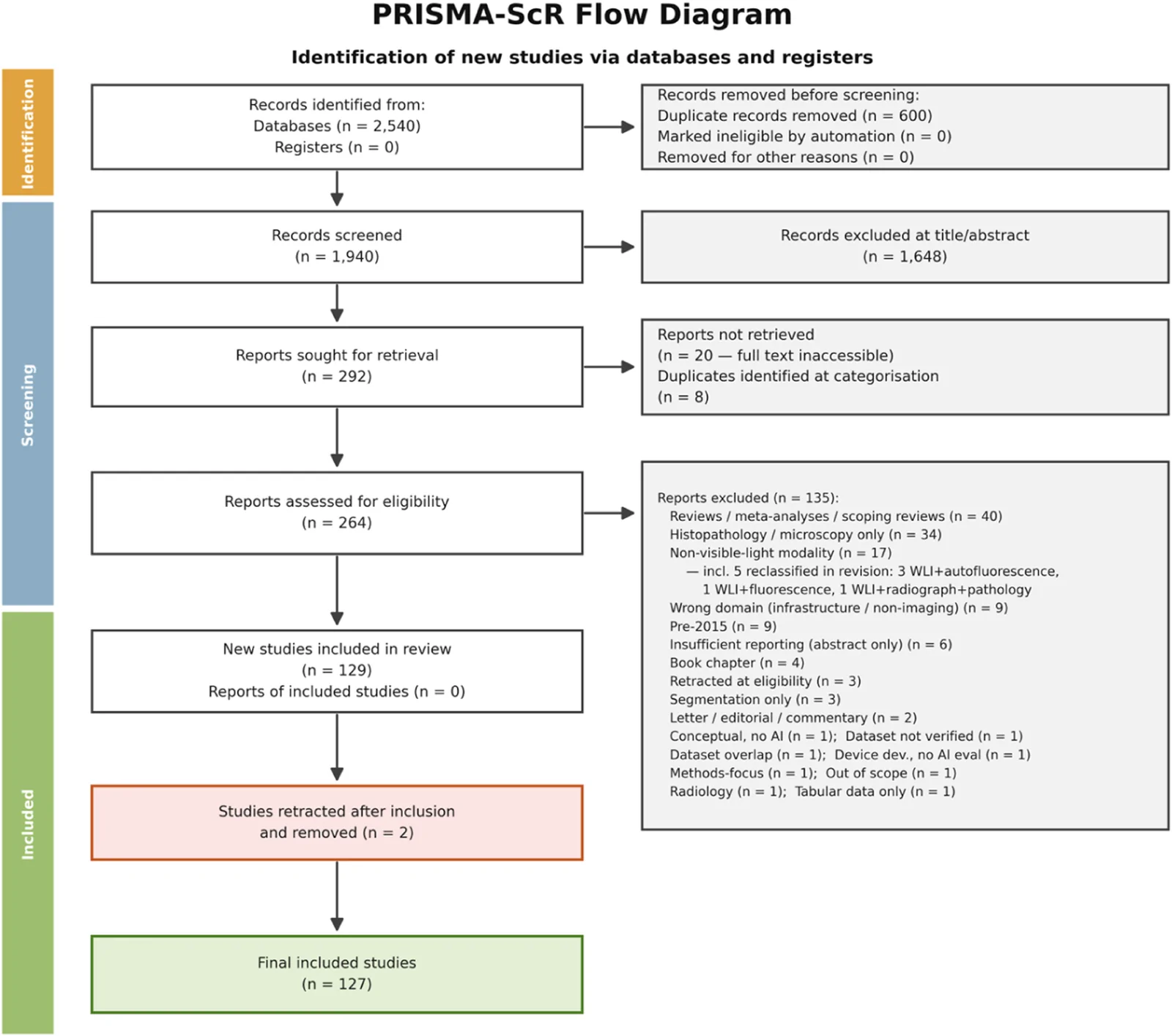
PRISMA diagram showing identification and screening stages with final included study count.

The search yielded 2,540 records, which were imported into Rayyan AI review software and reduced to 1,940 after deduplication.^24^ Title and abstract screening were conducted by a single reviewer (CG) against the pre-specified eligibility criteria (Table 1) resulting in 292 records progressing to full-text screening. Full-text screening was conducted in Zotero.^25^ (v7.0.30), also by a single reviewer (CG), against the same eligibility criteria, resulting in 127 included studies; no second author verification was conducted at any stage. Independent dual-reviewer screening, formal calibration exercises, and inter-rater agreement statistics were not undertaken; the implications for review reproducibility are acknowledged in the Limitations section. Figure 2 outlines the selection process and Table 1 outlines the inclusion/exclusion criteria.

**Table 1.** Scoping review inclusion and exclusion criteria.

Category	Inclusion criteria	Exclusion criteria
Study Objective	Studies that classify, or that classify AND detect, or predict oral potentially malignant disorders (OPMDs), dysplasia, or oral cancer	Studies focused solely on segmentation, feature extraction, or image enhancement without diagnostic or classification intent.
Study Type & Design	Original research articles using Artificial Intelligence (AI), Machine Learning (ML), or Deep Learning (DL). Peer-reviewed journal articles or conference papers published from 2015–2025.	Systematic reviews, meta-analyses, narrative reviews, editorials, or letters without new experimental results. Descriptive clinical studies or rule-based expert systems without ML/DL.
Population	Human subjects with oral lesions or oral cancer.	Studies using only animal data. Studies involving non-oral regions (e.g., skin, larynx, systemic cancers).
Data Modality	Must use clinical photographic images (RGB/visible light) taken of intraoral tissue (e.g., DSLR, smartphone, or intraoral camera).	Studies using non-visible-light modalities (e.g., autofluorescence, OCT, NIR, hyperspectral, radiography, histopathology, MRI). Studies based purely on textual EHR, genomic data, or pathology slides without contributing photographic image data.
Multimodal Inputs	Studies combining visible-light photographs with non-image data (demographics, clinical history, lesion site, textual metadata) are included; the non-image data are treated as multimodal inputs accompanying the primary imaging modality.	N/A
AI Methodology	Any algorithmic approach including traditional ML, CNNs, Vision Transformers, multimodal fusion, or hybrid systems (image + text/metadata).	N/A
Reporting Standards	Quantitative performance metrics must be provided (e.g., accuracy, sensitivity, specificity, AUC, F1).	Studies with unavailable performance metrics, unclear image modality, or no dataset description.
Language & Access	English-language publications with full text available (preprints acceptable if methodologically detailed).	Non-English publications.

Studies were included if they applied AI methods to visible-light intraoral clinical photographs as the primary or contributing imaging modality, and if lesion classification was the primary or substantive output. Multimodal studies - those combining photographs with non-image data such as demographics, lesion site, or clinical history - were included on the basis that visible-light imagery contributed to the input pipeline; non-image data were treated as accompanying multimodal inputs rather than as independent eligibility targets. Studies using fused non-visible-light imaging modalities (autofluorescence, OCT, hyperspectral, radiography, histopathology imaging) or only textual/structured clinical data without contributing photographic data were excluded.

English-language was a practical constraint of the review team. Conference proceedings were included given the rapid pace of AI methodology development in this domain - much of the recent technical work appears at IEEE/ACM and similar conference venues before formal journal publication, and excluding such work would systematically under-represent the field’s current direction. Preprints were admitted only where methodological reporting met the same data extraction standard applied to peer-reviewed studies.

Data extraction was performed using a structured template developed iteratively during a pilot phase (Supplementary File A), which defined each extraction field and its permissible categorical values; extraction categories used pre-specified mutually exclusive values for validation approach, learning paradigm, model architecture family, clinical task, and multimodal fusion strategy.

Where individual studies did not report a particular variable, the field was recorded as ‘NR’ (not reported), the proportion of NR responses for each variable is reported in Supplementary File B. Where reporting was ambiguous, the reviewer referred to the operational definitions in the extraction template, and the most specific applicable category was assigned, which was applied uniformly. A methodological characteristic rubric was applied to assess class imbalance, categorised as low, moderate, high, or unclear against pre-specified thresholds documented in Supplement File A. Class-imbalance categorisation refers to the smallest-class proportion of the total dataset: >25% is coded low imbalance, 10–25% moderate, and <10% high; studies whose class distribution was not reported in sufficient detail to compute the minority-class proportion were coded unclear.

Ground-truth label provenance was binary coded: study datasets were classed as histopathologically confirmed only if every output class carried lesion-level histological labels; all other configurations, such as malignant-only biopsy with clinical labelling of non-malignant classes, partial coverage, clinical-only labels, public-dataset use without documented provenance, and unreported provenance - were grouped as not fully confirmed. Histopathology with haematoxylin-and-eosin grading remains the conventional reference standard for oral lesion diagnosis.^26^

Each study’s task type was assigned to one of five mutually exclusive categories. The first four form an ordered progression reflecting depth of engagement with the dysplasia-to-cancer clinical pathway:

1. cancer endpoint only;
2. bundled-suspicious screening (OPMD and malignancy collapsed into one output class);
3. OPMD-aware (OPMD preserved as a discrete output category in any combination with benign or malignant);
4. dysplasia-engaged (within-OPMD risk stratification or explicit dysplasia/OED output classes); and
5. Residual - studies whose output was lesion-morphology only.

OPMD was kept distinct from histological epithelial dysplasia as an OPMD may or may not contain dysplastic change. For auditability, study assignment labels are available in Supplementary File B.^6^

Architectures were classified into two non-mutually-exclusive binary indicators derived from the extracted architecture type field and the primary model name: (i) CNN-based — the extracted architecture type included a convolutional designator or the primary model corresponded to a known CNN family (ResNet, DenseNet, VGG, Inception, EfficientNet, MobileNet, AlexNet, Xception, ConvNeXt, YOLO, Mask R-CNN, U-Net, or related variants); and (ii) transformer-based - the architecture type included “Transformer” or the primary model referenced ViT, Swin, DeiT, MobileViT, DETR, or a large foundation model (ChatGPT, GPT-4, Gemini). The two indicators are not mutually exclusive; studies whose pipeline incorporated both architectures contribute to both. Performance metrics were extracted as a single best-model record per study; per-architecture performance comparisons were therefore not derivable from the extraction and are not reported. Reporting completeness for each performance metric is reported in the Results section and detailed in Supplementary File B.

Validation was categorised by distinguishing internal validation from external validation on independent datasets collected at different institutions or time periods.^27^ Multimodal integration approaches combining imaging with clinical data were documented separately as were explainability.^28–30^ All extracted variables were recorded in Microsoft Excel file, where frequencies and graphs were also generated. A Claude AI model (Anthropic) was used during manuscript preparation to assist with spelling, grammar, and readability.

## Results

In this review, 127 studies published between 2015 and 2025 were included.^31–157^ Studies excluded during the full text review stage are available in Supplementary File B alongside exclusion rationales.

### Datasets and modalities

A single open-source dataset named “Oral Cancer (Lips and Tongue) Images” (OCI), 131 photographs (87 cancer/44 non-cancer) distributed without patient-level metadata and without histological ground-truth labelling - served as the data source for 27 of 127 studies (21.3%).^158^ Because it carries no patient identifiers, image-level random splitting was the only structurally available partitioning strategy in these studies, precluding patient-level separation; augmentation was used to expand the 131-image source in 7 of 27 studies, and external validation was reported by one. This collection is separate to a similarly named Kaggle dataset (“Oral Cancer Dataset”; 500 cancer/450 non-cancer) which is also observed in the reviewed corpus.^159^

Several studies assembled larger private clinical collections, with 44,409 images the largest reported.^32^ Most studies (87.4%, *n*=111/127) used clinical photographs as the sole or primary modality; 12.6% (*n*=16/127) used a multimodal approach combining clinical photographs with additional data types. Histopathological confirmation of lesion labels was reported in 7.9% (*n*=10/127); the remaining studies either partially reported confirmation for select classes or did not report histopathological ground truth at all. Among 16 studies using multimodal data, fusion strategy was specified in 13 and categorised as early fusion (*n*=5), intermediate-level fusion (n=6), or late fusion (n=2).

### Learning paradigms & tasks

Machine learning approaches can be categorised into supervised, unsupervised, semi-supervised, self-supervised and reinforcement learning.^160^ One study used a semi-supervised training approach; a multi-scale random cropping self-training framework for oral cancer versus leukoplakia classification, with a reported accuracy of 71.7%. The remaining studies used fully labelled datasets for training, no studies in the reviewed literature reported self-supervised, weakly supervised, or unsupervised learning approaches.^70^

One study used a supervised few-shot approach via a Siamese network producing matching scores for images across classes with reported 92% accuracy.^130^ Another was identified as having used synthetic oral lesion images generated through diffusion model–based inpainting synthesis to augment training data, achieving a reported diagnostic accuracy of 97%.^111^

**Figure 3.**
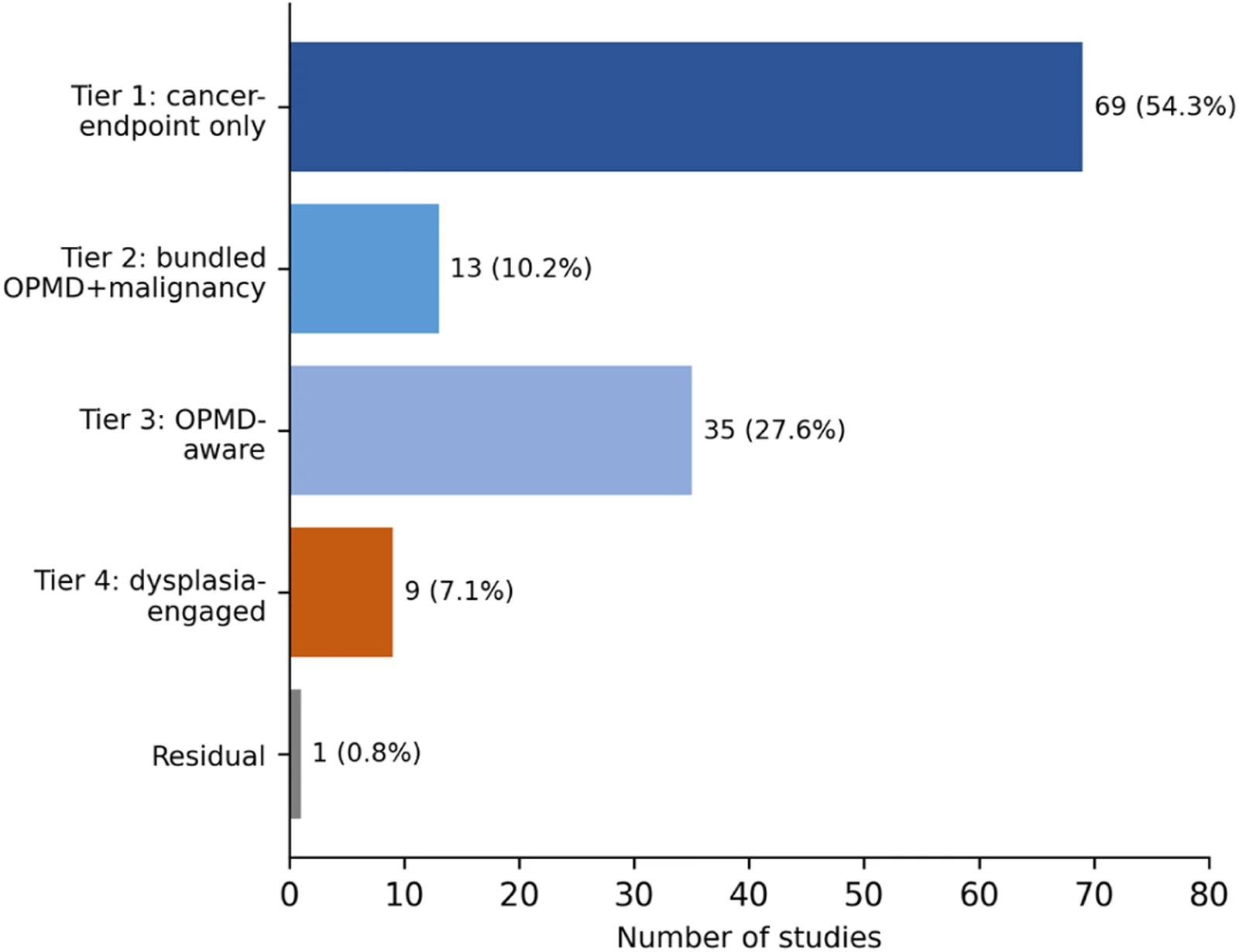
Distribution of the 127 included studies across five mutually exclusive categories - four ordered tiers of engagement with the benign–OPMD–malignant disease spectrum plus a residual category - defined by model output classes. The residual category comprises one study (0.8%) whose output was lesion-morphology only.

All 127 performed classification tasks, and not mutually exclusively, 16.5% (n=21/127) performed object detection and 7.1% (n=9/127) semantic segmentation to localise lesions. Across the included corpus, categorisation tasks can be distributed into five mutually exclusive tiers of engagement across the disease spectrum (Figure 3): (1) 54.3% (n=69/127) distinguished cancer from non-cancer only; (2) 10.2% (n=13/127) collapsed OPMDs and malignancy into a single “high-risk” class against benign or “low-risk” tissue; (3) 27.6% (n=35/127) preserved OPMDs as a discrete output category via three-way benign/OPMD/malignant classification, multi-lesion taxonomies, or OPMD-versus-malignancy comparisons; and (4) 7.1% (n=9/127) stratified risk within OPMDs or predicted epithelial dysplasia (OED) severity. A single residual study (0.8%) produced lesion-morphology output only.

Within the fourth tier one study collapsed the WHO mild/moderate/severe scale into a binary Class I/Class II risk classification, whereas other studies utilised coarser clinical proxies (low- versus high-risk OPMD, homogeneous vs non-homogeneous leukoplakia, or binary OED-vs-non-OED).^126^

### Preprocessing and validation

Data augmentation techniques were reported in 65.4% of studies (*n*=83/127), among these studies individual augmentation techniques were not mutually exclusive. Image flipping and rotation were each reported in 47.2% (*n*=60/127) and 48.0% (*n*=61/127) of studies respectively. Translation and scaling were each reported in 18.1% (*n*=23/127) and 19.7% (*n*=25/127) respectively, and brightness alterations in 14.2% (*n*=18/127). Gaussian noise (random pixel-level variation causing image degradation) was reported in 5.5% (*n*=7/127). Regarding class imbalance, of 127 included studies, 42.5% (n=54/127) were considered balanced, 18.9% (n=24/127) showed moderate imbalance, 15.0% (n=19/127) showed high imbalance, and 23.6% (n=30/127) could not be assessed from study reporting.

Segmentation techniques to isolate regions of interest were reported in 43.3% of studies (*n*=55/127), these were also non-mutually exclusive. Where the specific segmentation method was specified (*n*=52), the most common approach was manual region-of-interest annotation (*n*=32); automated segmentation with U-Net (*n*=5) or YOLO (*n*=3) architectures were reported less frequently.

Out of all 127 included studies, internal validation was reported in 91.3% (*n*=116/127). K-fold cross-validation was used in 17.3% (*n*=22/127). External validation was reported in 8.7% (*n*=11/127) of studies; and of these 6 used data from a different institution in the same country; 4 used data from a different country; and 5 used publicly available or internet-curated images as their external set. Categories overlap because several studies used multi-step strategies.

A multi-centre validation design, whereby model performance was evaluated across data from ≥2 independent institutions, was reported in 2.4% (*n*=3/127). These approaches were not mutually exclusive, for instance, studies reporting external validation typically also reported internal validation. Of the 127 studies, this review found 10.2% (*n*=13/127) to explicitly describe patient-level data splitting across training and test datasets. Image-level splitting was described explicitly by 18.1% (*n*=23/127) and the remaining 71.7% (*n*=91/127) did not explicitly define splitting method by either patient or image-level (Table 2).

**Table 2.** Methodological characteristics and reporting completeness across the 127 included studies. Categories are not mutually exclusive.

Methodological item	*n*	% of *n*=127
**Validation approach**		
Internal validation reported	116	91.3%
K-fold cross-validation	22	17.3%
External validation (any)	11	8.7%
Multi-centre validation (≥2 institutions)	3	2.4%
**Data splitting**		
Patient-level split (strict, explicit)	13	10.2%
Image-level split (explicit)	23	18.1%
Splitting method not reported	91	71.7%
**Performance metrics**		
Accuracy reported	108	85.0%
Sensitivity reported	102	80.3%
F1-score reported	83	65.4%
Specificity reported	56	44.1%
AUC/AUROC reported	39	30.7%
Confidence intervals reported	21	16.5%
**Deployment items**		
Clinical deployment discussed	119	93.7%
Image acquisition device named	61	48.0%
Human-expert comparison	18	14.2%

### Model architectures & performance

**Figure 4.**
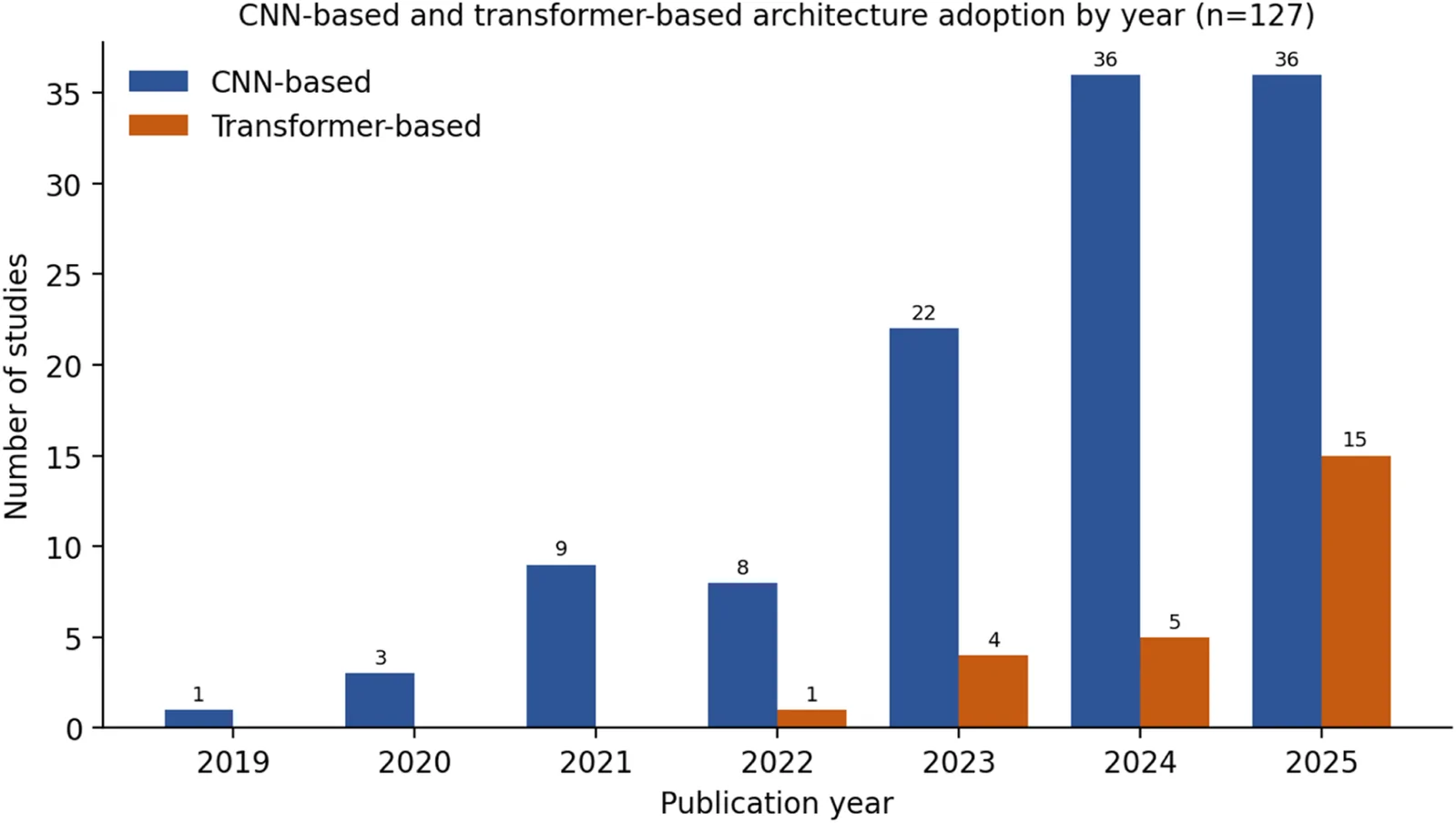
CNN-based and transformer-based architecture adoption across the 127 included studies, by publication year. Categories overlap where studies combine both components within a single pipeline.

Model architecture refers to the structural design of neural networks, determining how information flows through computational layers. Studies primarily used pre-trained convolutional architectures with transfer learning; a training strategy whereby models pre-trained on large-scale image datasets are further trained on domain-specific medical images. CNN-based architectures were most prevalent, reported in 90.6% (*n*=115/127) included studies; transformer-based architectures were reported in 19.7% (*n*=25/127) and emerged primarily from 2024 onwards (Figure 4). Architecture groups were not mutually exclusive; 13.4% (*n*=17/127) studies combined CNN and transformer components within a single pipeline. A full breakdown of specific architecture families encountered in either primary or comparator roles is provided in Supplementary File B.

Performance reporting across the 127 included studies was heterogeneous and incomplete. Accuracy was reported by 85.0% (*n*=108/127), sensitivity by 80.3% (*n*=102/127), F1-score by 65.4% (*n*=83/127), specificity by 44.1% (*n*=56/127), and AUC/AUROC by 30.7% (*n*=39/127). Confidence intervals were reported in 16.5% (*n*=21/127) of studies.

### Explainability approaches

Explainable AI (XAI)^161^ methods are designed to communicate the rationale behind model predictions. In the reviewed literature 37.0% (*n*=47/127) of studies described at least one XAI method. Among the 47 studies, individual techniques were not mutually exclusive: Grad-CAM was the most frequently reported approach (n=31, representing 24.4% of all 127 studies). Grad-CAM utilises gradients flowing into the final convolutional layer to highlight image regions identified as important by the model and was typically used in the reviewed literature to illustrate regions of model focus.^162^

Other XAI techniques reported, with their counts as a proportion of all 127 studies, were: saliency maps (2.4%, *n*=3), attention maps (3.9%, *n*=5), SHAP (2.4%, *n*=3) and LIME (0.8%, *n*=1). Two studies leveraged Case-Based Reasoning to provide more physician-aligned decision support and explainability of model predictions (Table 3).^61,105,153,161,163,164^

**Table 3.** A table showing AI explainability approaches across the cohort of included studies, categories are non-mutually exclusive.

XAI technique	*n*	% of *n*=127
Any XAI method reported	47	37.0%
Grad-CAM	31	24.4%
Attention maps	5	3.9%
Saliency maps	3	2.4%
SHAP	3	2.4%
LIME	1	0.8%
Case-Based reasoning	2	1.6%

### Deployment reporting

Reporting of deployment-relevant items varied across the 127 included studies. Clinical deployment was discussed in 93.7% (*n*=119/127) while the image acquisition device type was named in 48.0% (*n*=61/127) and human-expert comparison was reported in 14.2% (*n*=18/127). No included study reported a prospective evaluation of the AI model in a clinical workflow, although one study collected its data under a registered prospective protocol and evaluated the model post-hoc on the resulting dataset.^69^

## Discussion

The field of oral cancer classification using AI has extended rapidly over the past decade. However, compared to parallel areas of clinical image analysis, real-world deployment is lagging. In dermatology, for example, advances in vision models have started to make their way into real world clinical workflows.^165^

The task-design distribution of the included literature helps frame the gap between current AI capability and clinical utility. Most studies operate at a cancer-endpoint level only; asking the model to distinguish malignant from non-malignant. Far fewer preserve OPMD as a discrete output category, and within this group, three-way benign/OPMD/malignant classification is relatively uncommon. The implication is that the included research converges on a binary label structure (cancer vs not), while tasks which may hold greater utility in clinical practice, such as discriminating lesions with high vs low risk for dysplasia and malignant transformation, are less commonly modelled.

This may in part be explained by the challenges in assembling large oral image databases, potentially influencing researchers to collapse classification tasks into a binary setting. Anatomical constraints can make adequate photography difficult without mirrors and specialist equipment. This is compounded by data privacy concerns; with the face, teeth and oral features considered partly, if not fully identifiable. Methods aimed at resolving data privacy concerns include federated learning where AI models are trained on data at source, minimising data transfer between organisations.^111,166^ Moreover, the generation of synthetic images has been a suggested approach to addressing data scarcity - in this review we observed one study applying diffusion model–based inpainting synthesis to generate additional training images. Further studies into synthetic image generation within an oral health context would be needed to thoroughly outline the utility of synthetic images in model training and testing.

More broadly, multimodal data integration was reported in 12.6% of studies, with various fusion strategies reported. The methodological diversity within this subset is illustrated by one study utilising smartphone clinical photographs fused with patient age, sex, and habit data using early feature concatenation.^125^ Another study applied a customised multimodal deep learning framework (OMMT-PredNet) combining ResNet50 with convolutional block attention modules for image processing and a textual feature encoder for clinical variables, with feature concatenation, enabling both OED identification and time-dependent malignant transformation risk prediction.^126^ Whether multimodal approaches yield consistent improvements over image-only models would require comparative synthesis beyond the scope of this review.

In terms of training paradigms, no studies in the present review employed self-supervised learning (SSL) approaches; such as contrastive learning (SimCLR, MoCo), masked autoencoders, and self-distillation approaches (DINO).^167,168^ These techniques leverage large quantities of unlabelled images to learn visual representations. In fields such as radiology, suggested benefits of SSL focus on mitigating significant annotation burdens for specialist clinicians.^169^ Further investigation into SSL in the context of oral cancer is a recommendation of this review. However, as we have observed, the assimilation of datasets large enough to benefit from these approaches remain sparse.

Reflecting on model architectures, convolutional neural networks dominate the field by reported frequency across included studies, with ResNet-50 being the most frequently reported model overall. While CNNs excel at local feature extraction, they may be suboptimal for tasks requiring spatial reasoning across tissue regions in comparison to vision transformer (ViT) architectures in medical domains.^22^ We observed ViTs in the included paper corpus from 2024 onwards, and so far have demonstrated competitive performance in preliminary studies.^,42,54,69,72,90^ The emergence of vision-language foundation models represented a paradigm shift in the field of AI. These models, pre-trained on large-scale image–text pairs, can achieve competitive zero-shot performance on medical imaging tasks.^170,171^ Early studies evaluating multimodal large language models on oral lesion image classification show promise, though systematic benchmarking against purpose-built classifiers remains limited.^39,92,172^

Real-world deployment requires seamless integration with existing clinical information systems, decision support that aligns with clinical workflows, and outputs that support rather than replace clinical judgement. Currently, no studies have evaluated clinical workflow feasibility, time-to-decision impacts, or clinician acceptance of AI-assisted diagnosis. Further, real-world adoption will likely be heavily dependent on robust explainability frameworks.^29^ Gradient-weighted Class Activation Mapping (Grad-CAM) was the predominant explainability approach used across included studies. Advanced model-agnostic interpretability methods remained less common, however, with SHAP appearing in only 2.4% and LIME in 0.8% of studies. The integration of these approaches may reflect the rising emphasis of explainability in AI, but a wider plethora of techniques including intrinsic incorporation into model workflows remains open for exploration.

Temporal risk stratification and longitudinal monitoring represent underexplored applications with substantial clinical value. Oral lesions require extended surveillance periods, with transformation to malignancy occurring over months to years, yet most studies included in this review frame classification as single-timepoint task.^173^ One study, however, leveraged a baseline lesion photography with over 7 years of follow up records to identify time-to-event malignant transformation risk using a multimodal deep learning system, with a C-index of 0.79.^126^ This suggests that longitudinal multimodal data, and AI systems capable of integrating sequential data to model disease progression and predict transformation risk could potentially improve clinical management of OPMDs. Further investigations would be highly valuable to the field.

## Future directions and recommendations

To accelerate the safe and effective use of AI for classifying oral cancer and precursor lesions from visible light photography, several methodological and translational priorities should be addressed. First, curation of large, open, and well-annotated datasets must be a central goal. Datasets should capture real-world heterogeneity in acquisition devices, illumination conditions, anatomical sites, and disease spectra (normal mucosa, potentially malignant disorders, dysplasia grades, and invasive cancer). Standardised annotation protocols - ideally including lesion boundaries, uncertainty labels, and longitudinal outcomes - would enable robust benchmarking, reproducibility, and fair comparison across models.

Second, advanced image segmentation should be treated as a foundational step rather than an optional pre-processing task. Accurate delineation of lesions and surrounding mucosa can reduce background bias, improve feature localisation, and enhance interpretability. Contemporary approaches such as attention-guided segmentation, weakly supervised and self-supervised segmentation can leverage limited pixel-level labels while remaining robust to annotation noise. Joint optimisation of segmentation and classification in end-to-end frameworks may further improve diagnostic performance.^174–176^

Third, data augmentation and synthesis require more principled development beyond basic geometric or photometric transformations. Domain-aware and attribute-aware augmentation - accounting for colour constancy, illumination variability, specular highlights, and anatomical plausibility - is critical in oral imaging.^177,178^ Generative models, including diffusion-based synthesis and conditional generative adversarial networks, offer promising avenues to enrich minority classes, simulate rare precursor lesions, and improve model generalisation, provided that safeguards against artefact learning and distribution shift are rigorously applied.^179^

Fourth, class imbalance remains a defining challenge, as early-stage dysplasia and certain precursor lesions are under-represented relative to advanced disease or normal tissue. Addressing this requires a combination of strategies, including cost-sensitive, foca and asymmetric loss functions, informed resampling, and uncertainty-aware training.^180–184^ Importantly, evaluation metrics should prioritise clinical relevance - such as sensitivity for high-risk lesions and balanced accuracy - rather than overall accuracy alone.

Fifth, model fusion and ensemble learning should be systematically explored. Combining complementary representations - such as convolutional features, transformer-based global context, and graph-based relational modelling of lesion morphology - can improve robustness and reduce variance. Hybrid fusion strategies, including late-decision fusion, tailored to majority and minority classes, may be particularly effective in clinically imbalanced settings and better reflect multi-factorial clinician reasoning.^30,185^

Sixth, XAI is essential for clinical adoption. Models should provide transparent and reliable explanations at both the pixel and concept levels, highlighting diagnostically meaningful regions and features rather than spurious correlations. Techniques such as attention visualisation, counterfactual explanations, and concept-based attribution can support clinician trust, facilitate error analysis, and enable regulatory scrutiny.^186–188^ XAI should be evaluated not only for visual plausibility but also for clinical validity and consistency across patient subgroups.

Seventh, vision–language models (VLMs) represent a promising direction for future research in oral cancer and precursor lesion classification from visible light photography.^189^ By jointly learning from images and textual information, VLMs would enable the integration of visual lesion characteristics with clinical descriptors such as lesion morphology, anatomical site, risk factors, and provisional diagnoses. This multimodal representation may be particularly advantageous for early-stage and potentially malignant disorders, where visual features are subtle and subject to high inter-observer variability. Moreover, VLMs support weakly supervised, prompt-based, and few-shot learning paradigms, potentially reducing dependence on large, fully annotated datasets. Importantly, language grounding also offers a pathway toward more interpretable and clinically meaningful explanations, complementing pixel-level saliency methods. Future work should focus on domain-adapted VLM architectures tailored to intraoral imaging, robust handling of noisy or biased clinical text, and rigorous external validation to ensure generalisability and clinical trust.

Finally, future research should emphasise generalisation, fairness, and deployment readiness. External validation across institutions, devices, and populations is critical to mitigate bias and ensure equity. Prospective studies, human–AI interaction experiments, and integration with clinical workflows will be necessary to move from proof-of-concept models toward real-world impact. Collectively, addressing these issues will help ensure that AI systems for oral cancer and precursor lesion classification are accurate, interpretable, and clinically meaningful.

## Limitations

This scoping review has several limitations that should be considered when interpreting its findings. Together, the inclusion and screening choices described below introduce a risk of review-level selection bias. First, the search was limited to four databases (Scopus, Web of Science, Embase, and PubMed) and English-language publications, potentially missing relevant studies indexed elsewhere or published in other languages. Second, a single reviewer contributed to paper inclusions, which may limit bias mitigation. Third, conference papers were included to capture emerging research, which may introduce findings that have not undergone full peer review.

Fourthly, formal quality assessment and meta-analysis were not performed. Given substantial heterogeneity in study designs, datasets, and evaluation protocols pooled results may not be fully representative. Finally, publication bias likely inflates reported performance, as studies with negative or null findings may be underrepresented.

## Conclusion

This scoping review maps a field in which AI applied to visible-light intraoral photography has progressed rapidly on narrow benchmarks, with published performance figures frequently approaching ceiling on internal validation.

The areas where the reviewed literature is sparse - dysplasia grading, ordinal classification approaches and multimodal integration - may inform priorities for future research, though comparative evaluation would be needed to establish which approaches yield the greatest clinical benefit. Addressing these gaps will require collaborative efforts to develop larger, well-annotated datasets with histological validation, standardised evaluation protocols enabling meaningful comparison across studies, and clinical workflow integration studies that move beyond technical accuracy to assess real-world impact.

## Supplemental material

Supplemental material - A scoping review of AI in the classification of oral cancer and oral potentially malignant disorders from visible-light photography Supplemental material for A scoping review of AI in the classification of oral cancer and oral potentially malignant disorders from visible-light photography by Charles Goodmaker, Rishi Bhandari, Anwar Tappuni, Tuan D. Pham in Digital Health.

Supplemental material - A scoping review of AI in the classification of oral cancer and oral potentially malignant disorders from visible-light photography Supplemental material for A scoping review of AI in the classification of oral cancer and oral potentially malignant disorders from visible-light photography by Charles Goodmaker, Rishi Bhandari, Anwar Tappuni, Tuan D. Pham in Digital Health.

Supplemental material - A scoping review of AI in the classification of oral cancer and oral potentially malignant disorders from visible-light photography Supplemental material for A scoping review of AI in the classification of oral cancer and oral potentially malignant disorders from visible-light photography by Charles Goodmaker, Rishi Bhandari, Anwar Tappuni, Tuan D. Pham in Digital Health.

## Appendix

### Abbreviations

AI: Artificial Intelligence
AUC: Area Under the Curve
CNN: Convolutional Neural Network
CV: Computer Vision
EHR: Electronic Health Record
GAN: Generative Adversarial Network
Grad-CAM: Gradient-weighted Class Activation Mapping
HPV: Human Papillomavirus
IQR: Interquartile Range
LIME: Local Interpretable Model-agnostic Explanations
ML: Machine Learning
OCI: Oral Cancer (Lips and Tongue) Images dataset
OED: Oral Epithelial Dysplasia
OL: Oral Leukoplakia
OLP: Oral Lichen Planus
OPMD: Oral Potentially Malignant Disorder
OSCC: Oral Squamous Cell Carcinoma
OSMF: Oral Submucous Fibrosis
PRISMA-ScR: Preferred Reporting Items for Systematic Reviews and Meta-Analyses extension for Scoping Reviews
ResNet: Residual Network
ROI: Region of Interest
SD: Standard Deviation
SHAP: SHapley Additive exPlanations
UK: United Kingdom
VGG: Visual Geometry Group
ViT: Vision Transformer
WHO: World Health Organization
XAI: Explainable AI.
