# Supplementary material for: A scoping review of AI in the classification of oral cancer and oral potentially malignant disorders from visible-light photography
Source: Digit Health. 2026 Aug 22;12:20552076261481593. doi: 10.1177/20552076261481593 (PMC13500969; doi:10.1177/20552076261481593)
Supplement: Supplementary file 1 [file sj-pdf-1-dhj-10.1177_20552076261481593.pdf]

Supplementary Document

Scoping Review of AI for Oral Cancer and OPMD Classification on Visible-Light Photography

§A. Search strategy

Search terms (conducted on 28th October 2025 via web portals)

Scopus results returned = 1078

TITLE-ABS-KEY ((oral AND (cancer OR carcinoma OR OSCC OR dysplasia OR leukoplakia OR erythroplakia OR "potentially malignant" OR precancer OR OPMD OR OED))) AND TITLE-ABS-KEY (("artificial intelligence" OR "machine learning" OR "deep learning" OR "convolutional neural network*" OR cnn OR "neural network*" OR "computer vision" OR "transfer learning" OR "vision transformer" OR transformer OR resnet OR vgg OR densenet OR efficientnet OR "automated detection" OR "automated diagnosis" OR "automated classification" OR "computer aided diagnosis" OR "computer-aided diagnosis" OR "CAD system" OR "self-supervised" OR "few-shot" OR "federated learning" OR "multimodal AI")) AND TITLE-ABS-KEY ((photograph* OR photography OR image* OR imaging OR autofluorescence OR fluorescence OR optical OR "narrow band" OR NBI OR hyperspectral OR multispectral OR confocal OR "optical coherence tomography" OR OCT OR thermal OR infrared OR smartphone OR mobile OR "point-of-care" OR "image analysis" OR "image-based")) AND NOT TITLE-ABS-KEY ((radiograph* OR "CT scan" OR "computed tomography" OR MRI OR "magnetic resonance" OR "PET scan" OR "histopathology-only" OR "genomic-only")) AND PUBYEAR > 2014 AND PUBYEAR < 2026

WoS results returned 177

TS=(
("oral cancer" OR "oral cavity cancer" OR "oral squamous cell carcinoma" OR "OSCC"
OR "oral epithelial dysplasia" OR "OED" OR "oral potentially malignant disorder*"
OR "OPMD" OR "oral leukoplakia" OR "erythroplakia" OR "oral precancer" OR "oral mucosa")
AND
("artificial intelligence" OR "machine learning" OR "deep learning"
OR "convolutional neural network*" OR "CNN" OR "neural network*" OR "computer vision"
OR "transfer learning" OR "vision transformer" OR "ResNet" OR "VGG" OR "DenseNet"
OR "EfficientNet" OR "automated detection" OR "automated diagnosis"
OR "computer-aided diagnosis" OR "self-supervised learning"
OR "few-shot learning" OR "federated learning" OR "multimodal AI")
AND
("clinical photograph*" OR "intraoral image*" OR "oral image*" OR "oral imaging"
OR "smartphone imaging" OR "autofluorescence" OR "fluorescence imaging"
OR "optical imaging" OR "narrow band imaging" OR "hyperspectral imaging"
OR "multispectral imaging" OR "confocal microscopy"

OR "optical coherence tomography" OR "OCT" OR "thermal imaging"
OR "infrared imaging" OR "image analysis")
NOT
("radiograph*" OR "CT scan" OR "computed tomography" OR "MRI" OR "magnetic resonance"
OR "PET scan" OR "histopathology-only" OR "genomic-only")
)

embase results returned 916

('oral cancer':ti,ab,kw OR 'oral cavity cancer':ti,ab,kw OR 'oral squamous cell carcinoma':ti,ab,kw OR 'oscc':ti,ab,kw OR 'mouth cancer':ti,ab,kw OR 'tongue cancer':ti,ab,kw OR 'oral epithelial dysplasia':ti,ab,kw OR 'oed':ti,ab,kw OR 'oral potentially malignant disorder':ti,ab,kw OR 'oral potentially malignant disorders':ti,ab,kw OR 'opmd':ti,ab,kw OR 'oral leukoplakia':ti,ab,kw OR 'oral leucoplakia':ti,ab,kw OR 'erythroplakia':ti,ab,kw OR 'oral erythroleukoplakia':ti,ab,kw OR 'oral precancer':ti,ab,kw OR 'oral premalignant':ti,ab,kw OR 'oral mucosa':ti,ab,kw OR 'mouth tumor'/exp OR 'precancer'/exp) AND ('artificial intelligence':ti,ab,kw OR 'machine learning':ti,ab,kw OR 'deep learning':ti,ab,kw OR 'convolutional neural network':ti,ab,kw OR 'convolutional neural networks':ti,ab,kw OR 'cnn':ti,ab,kw OR 'neural network':ti,ab,kw OR 'neural networks':ti,ab,kw OR 'computer vision':ti,ab,kw OR 'transfer learning':ti,ab,kw OR 'vision transformer':ti,ab,kw OR 'transformer':ti,ab,kw OR 'attention mechanism':ti,ab,kw OR 'resnet':ti,ab,kw OR 'vgg':ti,ab,kw OR 'efficientnet':ti,ab,kw OR 'densenet':ti,ab,kw OR 'inceptionnet':ti,ab,kw OR 'mobilenet':ti,ab,kw OR 'ensemble learning':ti,ab,kw OR 'deep neural network':ti,ab,kw OR 'automated detection':ti,ab,kw OR 'automated diagnosis':ti,ab,kw OR 'automated classification':ti,ab,kw OR 'computer-aided detection':ti,ab,kw OR 'computer-aided diagnosis':ti,ab,kw OR 'computer aided diagnosis':ti,ab,kw OR 'cad system':ti,ab,kw OR 'support vector machine':ti,ab,kw OR 'random forest':ti,ab,kw OR 'gradient boosting':ti,ab,kw OR 'self-supervised learning':ti,ab,kw OR 'few-shot learning':ti,ab,kw OR 'federated learning':ti,ab,kw OR 'multimodal learning':ti,ab,kw OR 'artificial intelligence'/exp OR 'machine learning'/exp) AND ('clinical photograph':ti,ab,kw OR 'clinical photographs':ti,ab,kw OR 'clinical photography':ti,ab,kw OR 'intraoral photograph':ti,ab,kw OR 'intraoral image':ti,ab,kw OR 'intraoral images':ti,ab,kw OR 'intraoral imaging':ti,ab,kw OR 'oral photograph':ti,ab,kw OR 'oral image':ti,ab,kw OR 'oral images':ti,ab,kw OR 'oral imaging':ti,ab,kw OR 'visual examination':ti,ab,kw OR 'smartphone imaging':ti,ab,kw OR 'mobile imaging':ti,ab,kw OR 'point-of-care imaging':ti,ab,kw OR 'autofluorescence':ti,ab,kw OR 'fluorescence imaging':ti,ab,kw OR 'optical imaging':ti,ab,kw OR 'narrow band imaging':ti,ab,kw OR 'nbi':ti,ab,kw OR 'hyperspectral imaging':ti,ab,kw OR 'multispectral imaging':ti,ab,kw OR 'confocal microscopy':ti,ab,kw OR 'reflectance confocal microscopy':ti,ab,kw OR 'optical coherence tomography':ti,ab,kw OR 'oct':ti,ab,kw OR 'thermal imaging':ti,ab,kw OR 'infrared imaging':ti,ab,kw OR 'image analysis':ti,ab,kw OR 'image-based':ti,ab,kw OR 'photo-based':ti,ab,kw OR 'photograph-based':ti,ab,kw OR 'visual assessment':ti,ab,kw OR 'diagnostic imaging'/exp) NOT ('radiograph':ti,ab,kw OR 'radiographic':ti,ab,kw OR 'ct scan':ti,ab,kw OR 'computed tomography':ti,ab,kw OR 'mri':ti,ab,kw OR 'magnetic resonance':ti,ab,kw OR 'pet scan':ti,ab,kw OR 'histopathology only':ti,ab,kw OR 'genomic':ti,ab,kw OR 'molecular':ti,ab,kw)

pubmed results returned 369

(("oral cancer"[Title/Abstract] OR "oral cavity cancer"[Title/Abstract] OR "oral squamous cell carcinoma"[Title/Abstract] OR "OSCC"[Title/Abstract] OR "mouth cancer"[Title/Abstract] OR "tongue cancer"[Title/Abstract] OR "oral epithelial dysplasia"[Title/Abstract] OR "OED"[Title/Abstract] OR "oral potentially malignant disorder"[Title/Abstract] OR "oral potentially malignant disorders"[Title/Abstract] OR "OPMD"[Title/Abstract] OR "oral leukoplakia"[Title/Abstract] OR "oral leucoplakia"[Title/Abstract] OR "erythroplakia"[Title/Abstract] OR "oral erythroleukoplakia"[Title/Abstract] OR "oral precancer"[Title/Abstract] OR "oral premalignant"[Title/Abstract] OR "oral mucosa"[Title/Abstract] OR "mouth neoplasms"[MeSH Terms] OR "precancerous conditions"[MeSH Terms])) AND (("artificial intelligence"[Title/Abstract] OR "machine learning"[Title/Abstract] OR "deep learning"[Title/Abstract] OR

"convolutional neural network"[Title/Abstract] OR "convolutional neural networks"[Title/Abstract] OR "CNN"[Title/Abstract] OR "neural network"[Title/Abstract] OR "neural networks"[Title/Abstract] OR "computer vision"[Title/Abstract] OR "transfer learning"[Title/Abstract] OR "vision transformer"[Title/Abstract] OR "transformer"[Title/Abstract] OR "attention mechanism"[Title/Abstract] OR "ResNet"[Title/Abstract] OR "VGG"[Title/Abstract] OR "EfficientNet"[Title/Abstract] OR "DenseNet"[Title/Abstract] OR "InceptionNet"[Title/Abstract] OR "MobileNet"[Title/Abstract] OR "ensemble learning"[Title/Abstract] OR "deep neural network"[Title/Abstract] OR "automated detection"[Title/Abstract] OR "automated diagnosis"[Title/Abstract] OR "automated classification"[Title/Abstract] OR "computer-aided detection"[Title/Abstract] OR "computer-aided diagnosis"[Title/Abstract] OR "computer aided diagnosis"[Title/Abstract] OR "CAD system"[Title/Abstract] OR "support vector machine"[Title/Abstract] OR "random forest"[Title/Abstract] OR "gradient boosting"[Title/Abstract] OR "self-supervised learning"[Title/Abstract] OR "few-shot learning"[Title/Abstract] OR "federated learning"[Title/Abstract] OR "multimodal learning"[Title/Abstract] OR "artificial intelligence"[MeSH Terms] OR "machine learning"[MeSH Terms])) AND (("clinical photograph"[Title/Abstract] OR "clinical photographs"[Title/Abstract] OR "clinical photography"[Title/Abstract] OR "intraoral photograph"[Title/Abstract] OR "intraoral image"[Title/Abstract] OR "intraoral images"[Title/Abstract] OR "intraoral imaging"[Title/Abstract] OR "oral photograph"[Title/Abstract] OR "oral image"[Title/Abstract] OR "oral images"[Title/Abstract] OR "oral imaging"[Title/Abstract] OR "visual examination"[Title/Abstract] OR "smartphone imaging"[Title/Abstract] OR "mobile imaging"[Title/Abstract] OR "point-of-care imaging"[Title/Abstract] OR "autofluorescence"[Title/Abstract] OR "fluorescence imaging"[Title/Abstract] OR "optical imaging"[Title/Abstract] OR "narrow band imaging"[Title/Abstract] OR "NBI"[Title/Abstract] OR "hyperspectral imaging"[Title/Abstract] OR "multispectral imaging"[Title/Abstract] OR "confocal microscopy"[Title/Abstract] OR "reflectance confocal microscopy"[Title/Abstract] OR "optical coherence tomography"[Title/Abstract] OR "OCT"[Title/Abstract] OR "thermal imaging"[Title/Abstract] OR "infrared imaging"[Title/Abstract] OR "image analysis"[Title/Abstract] OR "image-based"[Title/Abstract] OR "photo-based"[Title/Abstract] OR "photograph-based"[Title/Abstract] OR "visual assessment"[Title/Abstract] OR "diagnostic imaging"[MeSH Terms])) NOT ("radiograph"[Title/Abstract] OR "radiographic"[Title/Abstract] OR "CT scan"[Title/Abstract] OR "computed tomography"[Title/Abstract] OR "MRI"[Title/Abstract] OR "magnetic resonance"[Title/Abstract] OR "PET scan"[Title/Abstract] OR "histopathology only"[Title/Abstract] OR "genomic"[Title/Abstract] OR "molecular"[Title/Abstract])

§B. Eligibility criteria

Refer to Table 1 of the main manuscript for the population-concept-context (PCC) eligibility framework. The summary below restates the inclusion / exclusion rules applied during screening:

- Inclusion: original empirical studies applying machine-learning or deep-learning models to visible-light intraoral photography for classification, detection, segmentation, or risk prediction of oral cancer or oral potentially malignant disorders (OPMDs).
- Visible-light photography had to be a substantive component of the model's input pipeline. Studies using histopathology images, autofluorescence imaging, hyperspectral imaging, OCT, radiography, or non-imaging molecular data as the sole input were excluded.
- Multimodal studies were included if visible-light photography was a primary input; demographics, clinical history, and lesion-site metadata were captured as secondary modalities where reported.
- Date range: 2015–2025. Language: English. Publication types: journal articles, conference papers, and preprints with adequate methodological reporting.
- Exclusion: systematic / scoping / narrative reviews, book chapters, letters to editor, conceptual papers without empirical evaluation, and papers without retrievable full text.

§C. Screening and verification workflow

Title/abstract and full-text screening were performed by a single reviewer (CG) using the eligibility criteria specified in §B and Table 1 of the main manuscript. To partially compensate for the absence of dual-reviewer screening, the following mitigations were applied:

- All studies, regardless of publication venue, were subject to the same 119-field structured data-extraction template (see §D).
- All 157 excluded reports (the count present in Sheet S2) were re-categorised post-hoc using a 19-category exclusion taxonomy. The categorised exclusions are provided in Supplementary File B, Sheet S2.

We acknowledge that single-reviewer screening does not permit calculation of inter-reviewer agreement statistics (e.g., Cohen's κ) and that this is a methodological limitation of the present scoping review, now also stated in the Limitations section.

§D. Data extraction template — field definitions

Domain	Example fields
Identification	Study ID, authors, year, journal, DOI, title
Study design	Study design type (retrospective / cross-sectional / experimental), validation approach (internal / external / multi-centre), splitting method, patient-level split
Dataset	Source, public yes/no, dataset name, total images, total patients, class distribution, image acquisition device, geographic location, collection period
Clinical task	Primary task (binary / multiclass / detection / segmentation), specific output classes, dysplasia / OPMD involvement, dysplasia grades or risk strata
Modality	Primary modality, multimodality flag, additional modalities, fusion strategy (early / feature-level / late / none)
AI methodology	Approach (DL / hybrid / traditional ML), architecture name, architecture family, pretrained yes/no, custom modifications
Preprocessing	Augmentation applied + techniques, segmentation applied + method
Performance	Accuracy, sensitivity, specificity, F1, AUC, confidence intervals reported, best-model performance summary
Interpretability	XAI method used, XAI technique, clinical interpretability discussed
Comparison	Baseline models compared, human-expert comparison
Risk of bias	Small sample, class imbalance, single-centre, no external validation, data leakage risk (each recoded low/moderate/high/unclear)
Clinical applicability	Deployment discussed, proposed use case, regulatory considerations, smartphone-based

§E. Risk-of-bias recoding rules

The five risk-of-bias indicators in our extraction template were originally coded as yes / no / unclear / NR. For the present analysis these were re-coded onto a low / moderate / high / unclear scale using the following rules (note we do not comment directly on ROB in the main manuscript instead we draw on data ingested from this original coding pipeline – this is because we found that ROB was difficult to reliable consolidate from the categories below alone and instead favoured commentary on each area individually):

RoB item	Rule

Small sample	N < 200 → high; 200–1,000 → moderate; > 1,000 → low; not reported → unclear
Class imbalance	Minority class < 10% → high; 10–25% → moderate; > 25% → low; cannot parse → unclear
Single-centre	Single source → high; 2–3 sources → moderate; multi-site or multi-country → low; unclear → unclear
No external validation	Internal-only → high; cross-validation / temporal only → moderate; external dataset → low; unclear → unclear

§F. Methodological-quality summary (Table S-X)

- Prospective AI evaluation: 0 (0%).

Items not captured as discrete extraction fields (code availability, data availability statements, calibration metrics, explicit predictive-uncertainty methods, target population specifics, decision threshold, failure modes, clinician acceptance) are flagged as 'not assessed in this review' and noted as a limitation.

§G. Stratified findings by dataset category (Table S-Y)

Per-study dataset and accuracy data are in Sheet S1b of Supplementary File B (the four-tier task stratification is derived from the task-tier field in Sheet S1b). Headline pattern (n=127):

Dataset category	n (% of 127)	External validation	Median accuracy [IQR]
Kaggle 131-image "Lips and Tongue" (OCI; def. + aug. + named)	27 (21.3%)	0 (0%)	96.2% [91.0–98.2]
Other Kaggle products (950-image variant, MOD, etc.)	5 (3.9%)	—	—
Other public dataset (non-Kaggle)	24 (18.9%)	—	—
Private clinically-curated	71 (55.9%)	—	86.5% [80.6–94.3]
Externally validated (overlapping)	11 (8.7%)	11 (100%)	89.3% [84.2–97.4]
Multi-centre (overlapping)	3 (2.4%)	3 of 3	—
All studies	127	11 (8.7%)	92.5% [84.8–97.5]

§H. External validation typology (Table S-AA)

Disaggregation of the 11 externally-validated studies (Sheet S5 of Supplementary File B; study IDs 6, 15, 29, 89, 90, 105, 108, 127, 129, 132, 134). Categories overlap because several studies used multi-step strategies, so the counts below sum to more than 11.

Type of external validation (categories overlap)	n	Notes

Different institution, same country	6	See per-study detail in Sheet S5
Different country / population	4	Cross-country validation remained rare
Publicly available / internet-curated external set	5	Several studies used multi-step strategies

§I. Patient-level split — audit summary

A manual audit of studies originally coded "patient-level split = yes" produced the final per-study classification below, which reconciles with the main-manuscript figures (strict patient-level 13 [10.2%]; explicit image-level 23 [18.1%]; not specified 91 [71.7%]). The audit reclassifications were (per-study splitting field in Sheet S1b of Supplementary File B):

- One-image-per-patient (1:1) ratio — not counted as strict patient-level per protocol; recoded to NR: 4 studies.
- Image-level split (re-coded from "yes" after methods review): 3 studies. Lesion-level clustering (same lesion may span train/test; not strict patient-level): 1 study (Patel et al. 2024).
- Not assessed (no PDF available): 1 study (Hamada et al. 2025). The remaining 114 studies (89.8%) were extracted as NR or explicit image-level.

§J. Deployment-relevant reporting (Table S-AB)

Binary reported / not reported summary for the deployment-relevant items captured in our extraction template (Sheet S1b of Supplementary File B (per-study deployment fields)).

Item	Reported	Not reported	Notes
Proposed clinical use case	127 (100%)	0 (0%)	Extracted
Image acquisition device named	61 (48.0%)	66 (52.0%)	Extracted
Histological / biopsy confirmation (all classes)	10 (7.9%)	117 (92.1%)	Manual second-round identification
Confidence intervals on metrics	21 (16.5%)	106 (83.5%)	Extracted (proxy for uncertainty)
Human-expert / clinician comparison	18 (14.2%)	109 (85.8%)	Extracted (proxy for workflow eval)
Prospective AI evaluation	0 (0%)	127 (100%)	Extracted; no prospective AI evaluations identified
Clinical deployment discussed (narrative)	119 (93.7%)	8 (6.3%)	Discussion-level only, not evaluation

Items requested by the reviewer but not captured as discrete extraction fields — target population specifics, decision threshold, calibration metrics, failure modes, clinician acceptance — are acknowledged as a limitation in the Limitations section, with a recommendation that these items be added to the extraction template in any future systematic review or living-review update.

§K. Reconciliation notes

Two figures in the manuscript were updated after the second-round audit; the differences are explained here for transparency.

Kaggle dataset reuse

- The 5 remaining studies in the broader "any Kaggle" group (any Kaggle = 32; 131-image OCI = 27; Sheet S4) used other Kaggle products, such as a ~950-image "Oral Cancer" variant and the Kaggle "Mouth and Oral Disease" multi-disease dataset.

Dysplasia involvement

Patient-level split

§L. Architecture reporting (Option A — added during revision)

During reviewer revision, architecture reporting in the main manuscript was simplified from family-level frequencies (under an any-mention rule) to two binary indicators only — CNN-based and transformer-based — to eliminate a unit-of-analysis mismatch between architecture counting (any-mention; primary + comparator) and performance reporting (best-model-per-paper).
Definitions:
• CNN-based: extracted architecture type includes "CNN" OR the primary model name corresponds to a known CNN family (ResNet, DenseNet, VGG, Inception, EfficientNet, MobileNet, AlexNet, Xception, ConvNeXt, YOLO, Mask R-CNN, U-Net, and related variants).
• Transformer-based: extracted architecture type includes "Transformer" OR the primary model name references ViT, Swin, DeiT, MobileViT, DETR, or a large foundation model (ChatGPT, GPT-4, Gemini).
• Categories are not mutually exclusive: a paper that uses both CNN and transformer components contributes to both indicators.

§M. Histopathological confirmation — re-extraction details

During revision, ground-truth label provenance was re-coded as a binary characteristic for the primary analysis: a study was classed as fully histopathologically confirmed only if every output class carried lesion-level histological labels. All other configurations were grouped as not fully confirmed. This collapses the original six-category extraction (retained as a secondary detail field in S7_Histo_Confirmation) and avoids load-bearing inference from small cells. Coding rule applies across all 127 included studies.
The secondary detail field uses the following five mutually exclusive sub-categories within the not-fully-confirmed group:
• histo_malignant_only — malignant (and/or OPMD) classes biopsy-confirmed; benign/normal classes clinical-only or unstated.
• histo_partial — mixed/partial coverage that does not fit the malignant-only pattern.
• clinical_only — labels assigned by clinical inspection, expert consensus, or biopsy without histological grading.
• public_dataset_no_provenance — public dataset used; label provenance not described.
• not_reported — paper silent on label provenance.

Flattened into binary coding post-hoc for readability and interpretation.

§N. Performance metric reporting (added during revision)

Performance reporting in the main manuscript was reframed during revision as a methodological-quality characteristic (reporting completeness) rather than synthesis of effect sizes. The unit of analysis is the single best-model record per study; per-architecture performance comparisons are not reported because of the unit-of-analysis mismatch between architecture counting (any-mention) and performance counting (best-model-only).

§O. 2026 revision — retractions, scope clarifications, and class-imbalance recompute

Retractions. Two studies included in the original analysis were retracted after inclusion and have been removed from all analyses (final n=127): Wang et al. 2023 (Sci Rep; 10.1038/s41598-023-49438-x; retracted 7 Oct 2025) and Zhang et al. 2024 (Heliyon; 10.1016/j.heliyon.2024.e32077; retracted). Both are recorded in Sheet S2 with the retraction rationale; all summary sheets in Supplementary File B were recomputed at n=127.

Modality scope. Oral white-light endoscopic images (one study, Heo et al. 2022) are treated as within the visible-light intraoral imaging scope of this review and retained; this is now stated explicitly in the main-text Methods. Two studies that pair clinical photography with a histopathology-microscopy dataset (Asif et al. 2025; Saxena et al. 2025) are retained on the basis that visible-light photography is a substantive model input; their histopathology-microscopy results are outside review scope and are excluded from the modality statistics.

Class-imbalance recompute. The class-imbalance indicator was recomputed directly from the extracted class-distribution counts against the rubric in §E (minority class <10% high; 10–25% moderate; >25% low; not parseable unclear). Twenty-three studies were recoded where the extracted counts contradicted the prior grade (SIDs 6, 7, 14, 29, 32, 34, 35, 40, 43, 49, 62, 70, 74, 79, 89, 91, 102, 103, 108, 113, 129, 131, 132). The revised distribution (n=127) is low 54 (42.5%), moderate 24 (18.9%), high 19 (15.0%), unclear 30 (23.6%). These recodings are flagged for co-author review.
